# Internal carotid artery fenestration with contralateral internal carotid artery dissection: a case report and literature review

**DOI:** 10.3389/fsurg.2026.1864348

**Published:** 2026-07-15

**Authors:** Dehong Yang, Zengjing Cheng, Yunjia Zhu, Lingxiang Fan, Binglin Chen, Chungang Dai, Ailin Chen, Qing Zhu

**Affiliations:** 1 Department of Neurosurgery, The Second Affiliated Hospital of Soochow University, Suzhou, China; 2 School of Pharmacy, Nantong University, Nantong, China; 3 Department of Neurosurgery, The Affiliated Suzhou Hospital of Nanjing Medical University, Suzhou Municipal Hospital, Suzhou, China

**Keywords:** arterial dissection, arterial fenestration, case report, hemodynamics, internal carotid artery

## Abstract

**Background:**

Internal carotid artery fenestration (ICAF) is a rare congenital cerebrovascular anomaly. Its coexistence with contralateral internal carotid artery (ICA) dissection is exceptionally uncommon, with only isolated case reports in the literature. The clinical features, hemodynamic characteristics, and optimal management of this rare combined lesion remain poorly understood. Conventional imaging alone cannot objectively evaluate lesion risk, so we report this case combined with hemodynamic analysis to clarify its pathogenesis and guide individualized treatment.

**Case presentation:**

We report a 71-year-old female who presented with episodic dizziness and had no history of hypertension or diabetes. Physical and routine laboratory examinations were unremarkable. Computed tomography angiography (CTA) suggested bilateral ICA C1 dissections. Digital subtraction angiography (DSA) confirmed a proximal fusiform dilation with intraluminal flow stratification (dissection) on the left C1 and a fenestration (two separate lumens with smooth intact walls) on the right C1. Computational fluid dynamics (CFD) analysis was performed to evaluate flow pressure (FP), wall shear stress (WSS), and oscillatory shear index (OSI). The left dissection showed significantly elevated FP, moderately elevated WSS at the entry and markedly elevated WSS at the exit, and high OSI. In contrast, the right fenestration demonstrated lower and uniformly distributed FP, relatively uniform WSS, and elevated OSI only in the smaller channel. Based on these findings, the left dissection was considered high-risk for rupture requiring active intervention, while the right fenestration was deemed hemodynamically stable and managed conservatively. The patient received dual antiplatelet therapy (aspirin 100 mg/d + clopidogrel 75 mg/d) for three days, followed by stent-assisted coiling of the left dissection. Her dizziness resolved completely after the procedure.

**Conclusion:**

Eight-month follow-up DSA confirmed complete resolution of the left dissection with preserved distal flow and stable morphology of the right fenestration. This case demonstrates that CFD hemodynamic analysis can help identify high-risk lesions and guide individualized treatment in patients with ICAF and contralateral dissection. Conservative management with antiplatelet therapy is appropriate for hemodynamically stable ICAF.

## 1 Introduction

Cerebral artery fenestration is a rare congenital cerebrovascular developmental anomaly, most frequently involving the basilar and vertebral arteries, with a population prevalence of approximately 2.1%. Internal carotid artery fenestration (ICAF) accounts for only 1.3% of cerebral artery fenestration cases (1), while its association with contralateral ICA dissection is exceedingly rare (2–6). At present, the treatment of ICA dissection at home and abroad tends to be consistent, that is, according to the different types of lesions, antiplatelet drugs can be given for conservative observation or endovascular stent implantation. For ICAF, no intervention is performed unless an aneurysm is induced or a source of intracranial emboli. However, the above treatment indications are symptom-oriented, mainly based on doctor ’s experience and imaging findings, and there is still a lack of relatively objective evaluation indicators. This article presents a case of ICAF combined with contralateral ICA dissection and discusses the pathological mechanisms and therapeutic strategies for these concurrent conditions through a literature review. According to the results of CFD hemodynamics, we more objectively evaluated the harm of these two lesions coexisting in this case, and could more accurately define the treatment indications.

## 2 Case presentation

**Figure 1:**
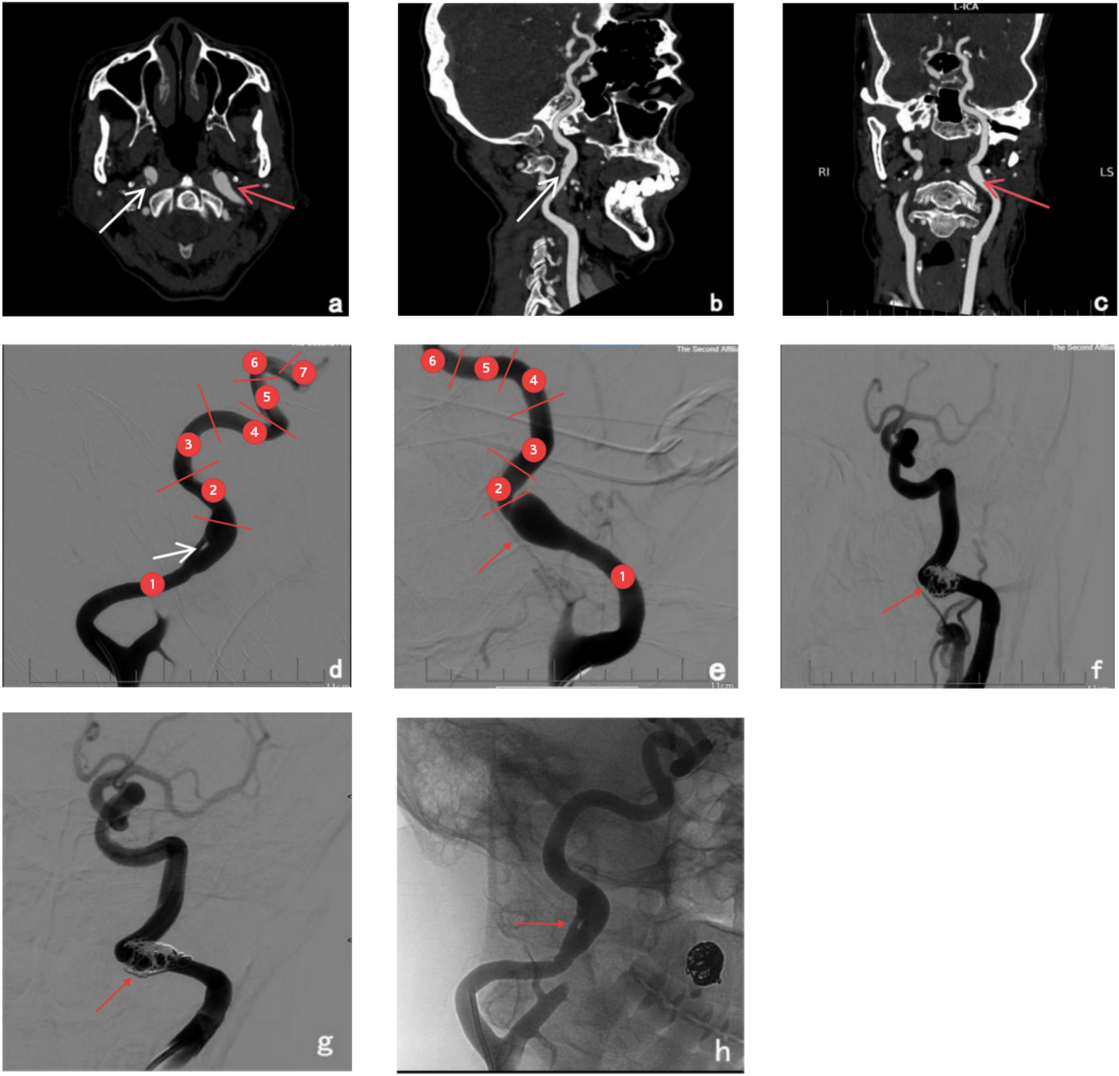
Preoperative and postoperative follow-up imaging. [The Bouthillier classification was adopted for internal carotid artery segmentation in Figure d, e (21)]. **a**: Contrast-enhanced CTA axial view demonstrates a “double lumen sign” in the right ICA (white arrow) and focal dilation of the left ICA (red arrow). **b**: CTA sagittal view reveals a linear filling defect in the right ICA C1 segment (white arrow). **c**: CTA coronal view shows focal dilation with distal narrowing in the left ICA C1 segment (red arrow). **d**: DSA (left anterior oblique projection) illustrates fenestration of the right ICA C1 segment, with two distinct but fully developed lumina of varying calibers (white arrow); The numbers represent different segments of the internal carotid artery, and the red lines indicate the approximate boundaries of each segment. **e**: Pre-embolization DSA (right anterior oblique projection) demonstrates the left ICA C1 segment dissection with patent distal flow (red arrow); The numbers represent different segments of the internal carotid artery, and the red lines indicate the approximate boundaries of each segment. **f**: Postoperative DSA (anteroposterior projection) confirms successful stent-assisted coil embolization of the left ICA C1 segment dissection and preserved distal perfusion (red arrow). **g**: Follow-up DSA at 8 months postoperatively shows complete resolution of the left ICA C1 segment dissection with no contrast filling and favorable parent artery remodeling (red arrow). **h**: Follow-up DSA at 8 months reveals stable morphology of the right ICA C1 segment fenestration compared to preoperative imaging (red arrow).

A 71-year-old female presented with episodic dizziness lasting over one month that relieved spontaneously after every episode lasting for several seconds. She had a 40-year history of alcohol abuse but no chronic conditions such as hypertension, diabetes, or cardiovascular disease. Physical examination and laboratory tests revealed no significant abnormalities, and neurological examination was unremarkable. Computed tomography angiography (CTA) performed at a local hospital suggested bilateral internal carotid artery (ICA) C1 segment dissections (Figures 1A–C). Subsequent digital subtraction angiography (DSA) at our institution demonstrated: Left ICA C1 segment: Proximal fusiform dilation (15 mm × 10 mm) with intraluminal blood flow stratification but preserved distal perfusion. Right ICA C1 segment: Fenestration at the proximal segment (Figures 1d,e). Three-dimensional rotational angiography (3DRA) was performed to acquire bilateral ICA images, which were reconstructed into Virtual Reality Modeling Language (VRML) files. These files were imported into ANSYS Fluent (ANSYS Inc., USA) for computational fluid dynamics (CFD) simulations. Key hemodynamic parameters—flow pressure (FP), wall shear stress (WSS), and oscillatory shear index (OSI)— were analyzed.

## 3 Computational fluid dynamics (CFD)

### 3.1 3D geometry reconstruction

During DSA, three-dimensional rotational angiography (3D-RA) was acquired for both ICAs, and raw DICOM data were exported. Reconstruction was performed using dedicated software (Syngo X-Workplace, Siemens). The region of interest (ROI) containing the left dissection and right fenestration was extracted separately and saved in STL format. All reconstructions were performed by an experienced neurointerventionalist to minimize operator bias.

### 3.2 Mesh generation

STL files were imported into ANSYS ICEM CFD (ANSYS Inc., USA). The vessel wall was meshed with triangular surface elements, and tetrahedral volume meshes were generated. Five layers of prismatic boundary layer elements were added near the wall to accurately capture velocity gradients, with a first layer thickness of 0.01 mm and a growth rate of 1.2. Grid independence was verified according to published criteria (9, 10). Final volume mesh sizes ranged from 1.5 to 2.0 million elements, with skewness <0.85 and orthogonal quality >0.15. The effect of mesh resolution on WSS and OSI calculations was considered, as OSI is more sensitive to grid refinement (11).

### 3.3 Boundary conditions

#### 3.3.1 Inlet

A velocity inlet boundary condition was applied based on preoperative transcranial Doppler (TCD) measurements and reported normal ICA flow velocities. A pulsatile velocity waveform over one cardiac cycle (0.8 s, heart rate 75 bpm) was imposed, with a mean velocity of 0.35 m/s and peak velocity of approximately 0.65 m/s, as previously characterized for the normal internal carotid artery (12).

#### 3.3.2 Outlet

A zero pressure outlet (relative pressure 0 mmHg) was used, coupled with a 3-element Windkessel model to simulate distal microcirculatory resistance. The parameters were set as Rc = 0.1 mmHg·s/mL, Rp = 1.2 mmHg·s/mL, and C = 1.0 mL/mmHg, following established protocols for cerebral hemodynamic simulations (13, 14). The influence of different outlet boundary conditions on WSS calculation has been quantitatively evaluated (7).

#### 3.3.3 Wall condition

Vessel walls were assumed rigid with no-slip condition, which is acceptable for comparing relative hemodynamic differences between lesions (8).

### 3.4 Blood properties

Blood was modeled as a Newtonian, incompressible fluid with density *ρ* = 1060 kg/m^3^ and dynamic viscosity *μ* = 0.0035 Pa·s (3.5 cP). This simplification has been validated for cerebral vasculature simulations (8).

### 3.5 Solver settings

Meshes were imported into ANSYS Fluent 2022 R1 (ANSYS Inc., USA). A pressure-based solver with the SIMPLEC algorithm for pressure-velocity coupling was used (15). Momentum, turbulent kinetic energy, and dissipation rate equations were discretized using second-order upwind schemes (16). Convergence criteria were set as residuals <1 × 10^−5^. Simulations were run for at least three cardiac cycles until flow parameters reached periodic stability.

### 3.6 Hemodynamic parameter extraction

The following parameters were calculated:

- Flow pressure (FP): Static pressure on the vessel wall (mmHg).
- Wall shear stress (WSS): *τ*_w = *μ* × (∂u/∂y)|_wall (Pa) 17].
- Oscillatory shear index (OSI): OSI=0.5 × [1 – (|∫₀ᵀ WSS·dt|/∫₀ᵀ |WSS| dt)], ranging from 0 (unidirectional steady flow) to 0.5 (fully oscillatory flow) (20, 21). OSI reflects directional instability of blood flow during a cardiac cycle, with higher values indicating turbulent flow (18, 20).

Time-averaged and peak values of FP, WSS, and OSI were extracted at the entry, exit, and dilated segment of the left dissection, as well as in both channels of the right fenestration, for comparative analysis.

#### 3.6.1 Hemodynamic findings and clinical decision

**Figure 2:**
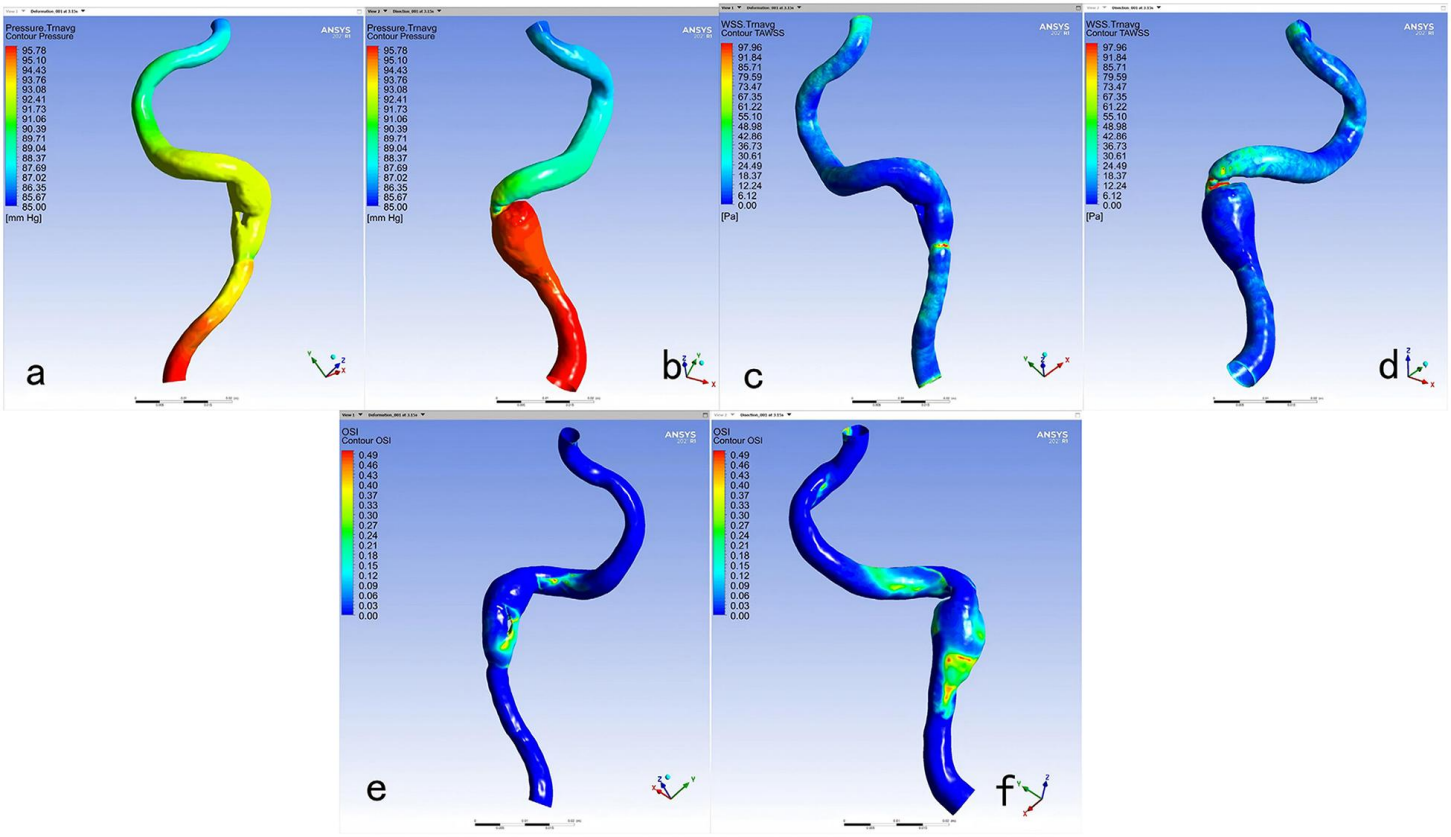
Comparative hemodynamic parameters of bilateral ICAs. **a,b**: Peak flow pressure (FP) on the dissection side significantly exceeded 95 mmHg (black arrow), while the fenestration side exhibited a peak FP of approximately 90 mmHg. **c,d**: Wall shear stress (WSS) at the dissection exit was markedly elevated compared to other regions, whereas WSS distribution across the fenestration side remained relatively uniform. **e,f**: Oscillatory shear index (OSI) was elevated within the dissection lumen. Notably, the smaller channel of the fenestration demonstrated higher OSI values compared to other regions.

These results suggested that, FP significantly higher on the dissection side compared to the fenestration side (Figures 2a,b). WSS Slightly elevated at the fenestration entry but generally uniform across the fenestration; markedly higher at the dissection exit vs. its entry (Figures 2c,d). OSI Elevated within the dissection lumen and in the smaller channel of the fenestration (Figures 2e,f).

The above results suggest, right ICA fenestration: No thrombotic signs, adequate perfusion, low FP, stable WSS, and minimal OSI suggested higher intervention risks than benefits. Left ICA dissection: Large aneurysm (15 mm × 10 mm), elevated FP, dynamic WSS gradients, and high OSI indicated substantial rupture risk, warranting aggressive intervention. Based on the above analysis results, the patient received dual antiplatelet therapy [aspirin 100 mg orally once daily (QD) + clopidogrel 75 mg QD] for 3 days prior to endovascular treatment. An Axium Detachable Coil (3D, 14 × 40 mm, Medtronic, USA) was deployed within the lumen of the dissecting aneurysm at the left internal carotid artery. An Enterprise stent (4.5 × 28 mm, Johnson & Johnson, USA) was implanted to cover the distal segment of the dissecting aneurysm, followed by the deployment of an Enterprise No Tip stent (4.5 × 28 mm, Johnson & Johnson, USA) to cover the proximal segment of the lesion. The procedure was completed with the additional embolization of another Axium Detachable Coil (3D, 14 × 40 mm, Medtronic, USA) into the aneurysm cavity. (Figure f). Postoperatively, dizziness resolved significantly within 2 days, and the patient was discharged with continued clopidogrel (75 mg QD for 6 weeks) and lifelong aspirin (100 mg QD). At 8-month follow-up, dizziness had completely resolved. Repeat DSA confirmed complete resolution of the left ICA dissection with preserved distal flow and stable right ICA fenestration (Figure g, f).

## 4 Discussion

ICAF is a rare cerebrovascular developmental anomaly with few reports in the literature. Its pathogenesis includes both embryogenic and acquired mechanisms. Failure of the primitive vascular plexuses originating from the third arch to fully regress and fuse may lead to C1 fenestration. Alternatively, abnormal connections between the C1 and the ascending C2, as well as persistent carotid canal, may also induce fenestration, with the latter emphasizing that vascular wall injury may result in pseudofenestration (4, 24, 38–42). We systematically reviewed all published case reports of ICA fenestration and summarized their clinical characteristics in Table 1. Several case reports have documented the coexistence of ICAF with contralateral ICA dissection. Ahn JY et al. reported the first case of ICA fenestration associated with arterial dissections, describing fenestration at the right distal C1-C2 with contralateral dissection; the dissection was treated with a stent while the fenestration was managed conservatively (27). Gailloud P et al. reported a case of traumatic right ICA fenestration accompanied by bilateral ICA dissections (4). More recently, Mărginean et al. presented a patient with right ICA fenestration initially misdiagnosed as dissection on CTA, underscoring the diagnostic challenge in distinguishing true fenestration from dissection (33). The present case is notable for several unique features: first, the use of CFD analysis to quantitatively compare hemodynamic profiles between the dissection and the contralateral fenestration in the same patient; second, the demonstration that hemodynamically stable ICAF can be safely managed with antiplatelet therapy alone; and third, the provision of detailed CFD methodology that may serve as a reference for future studies of similar rare vascular anomalies. To our knowledge, no prior report has employed patient-specific CFD to evaluate the differential hemodynamic risk between ICAF and contralateral dissection in the same individual.

**Table 1:** The case reports of ICA window opening that have been published to date.

Case Number	First author/Publication year	Age (years)/Gender	location	complication	Treatment	Clinical outcome	Imaging modality
1	Killien FC/1980 (22)	35/F	Left，C1–C2	-	Observation	No change	DSA
2	Yoichi Tanaka/1982 (23)	58/M	Right，C1–C2	ACoA	Observation	Death	DSA
3	Hasegawa T/1985 (2)	47/F	Right，C1，distal segment	The bifurcation of the right MAC aneurysm	Observation	Death	DSA
4		51/M	Right，C1，distal segment	Right temporal lobe malignant glioma	Observation	Improvement	DSA
5	Nakamura H/1993 (3)	71/M	Left，C1，distal segment	TIA	Antiplatelet	Improvement	DSA
6	Chess MA/1995 (24)	46/M	Right，C1–C2	Right-sided cervical squamous cell carcinoma	Observation	No change	DSA
7	Koenigsberg RA/1995 (25)	61/F	Right，C1–C2，distal segment	Right-sided tinnitus	Observation	No change	DSA
8	Mangla S/1999 (26)	73/M	Right，C1，distal segment	Parkinson's disease	Observation	No change	DSA
9	Ahn JY/2003 (27)	49/F	Right，C1–C2，distal segment	Left ICA dissection	Stent implantation + Antiplatelet	Improvement	DSA
10	Goto Y/2003 (28)	55/M	Right，C1，Proximal segment	tinnitus	Observation	No change	DSA
11		52/M	Left，C1，distal segment	TIA	Observation	No change	DSA + MR
12	Chng SM/2004 (29)	57/-	Right，C1，distal segment	-	Observation	No change	DSA + MR
13	Gailloud P/2004 (4)	59/F	Left，C1，distal segment	Stenosis of the right ICA	Observation	No change	DSA
14		52/M	Left，C1–C2，distal segment	Left basal ganglia region DVA	Observation	No change	DSA
15		51/F	Left，C1，Proximal segment	Left VA aneurysm, left C5 artery aneurysm	Observation	No change	DSA
16		74/M	Right，C1–C2，distal segment	FMD	Anticoagulation	Improvement	DSA
17		41/F	Right，C1，distal segment	FMD	Observation	No change	DSA
18		46/F	Right，C1，distal segment	Stenosis of the left ICA	Observation	Improvement	DSA + MR
19	Kung DK/2011 (30)	65/F	Left，C1，Whole segment	Right PCoA aneurysm	Stent implantation	Improvement	DSA
20	Hacein-Bey L/2013 (31)	59/F	Left，C1–C2，distal segment	-	Antiplatelet + Anticoagulation	Improvement	DSA
21	Liang ZG/2016 (32)	60/M	Right，C1，distal segment	-	Antiplatelet	Cured	DSA + MR
22	Zheng MM/2020 (6)	60/M	Left，C1，Whole segment	Left-sided watershed infarction	Stent implantation	Improvement	DSA
23	Mărginean L/2020 (33)	54/F	Right，C1，distal segment	FMD	Stent implantation	Recanalization	DSA
24	MNasel C/2022 (34)	62/M	Right，C1，distal segment	TIA	Antiplatelet	Improvement	DSA
25	WANG JW/2024 (35)	51/F	Right，C1，Midpiece segment	TIA	Antiplatelet	Improvement	MRI
26	Ezra Y Koh/2024 (36)	74/M	Bilateral, C1, distal segment	-	Observation	No change	CTA
27	Natalia V P/2024 (37)	47/F	Right，C1，distal segment	Pulsatile tinnitus	Observation	No change	CTA + DSA

ACoA, anterior communicating artery; MAC, middle cerebral artery; TIA,: transientischemic attack; DVA, developmental venous malformations; VA, vertebral artery; FMD, fibromuscular dysplasia; PCoA, posterior communicating artery.

### 4.1 Hemodynamic analysis and clinical decision-making

The present case offered a unique opportunity to compare hemodynamic parameters between a left ICA dissection and a contralateral true ICA fenestration in the same patient. CFD analysis revealed markedly different profiles across several key hemodynamic metrics, which directly informed the clinical decision to proceed with endovascular treatment for the dissection while adopting conservative management for the fenestration.

Flow pressure (FP) is a critical determinant of vascular wall stress, with elevated FP increasing the mechanical burden on the vessel wall and predisposing to wall injury, dissection propagation, and rupture. In intracranial aneurysms, high-pressure zones have been shown to correlate with the rupture point, suggesting that FP is a meaningful risk indicator (13, 43). In our patient, the left dissection exhibited peak FP exceeding 95 mmHg, significantly higher than the fenestration (approximately 90 mmHg), indicating substantially elevated wall stress that warranted active intervention.

Wall shear stress (WSS) represents the frictional force exerted by blood flow on the endothelial surface. Low WSS promotes pro-inflammatory gene expression, endothelial dysfunction, and accumulation of harmful metabolites, while elevated WSS can induce mechanical injury and accelerate inflammatory progression (44, 45). A particularly important finding in our case was the “bimodal” WSS pattern observed in the dissection: moderate elevation at the entry site and marked elevation at the exit site. This pattern is consistent with flow acceleration through a stenotic segment followed by jet impingement at the re-entry point, which may predispose to further wall injury, aneurysm formation, and eventual rupture. In contrast, the fenestration displayed relatively uniform WSS distribution without abrupt gradients, consistent with its stable clinical course and absence of thrombosis.

The oscillatory shear index (OSI) quantifies the directional instability of blood flow over a cardiac cycle, with higher values indicating turbulent or disturbed flow (18, 19). Elevated OSI has been consistently associated with pro-inflammatory endothelial activation, increased risk of thrombus formation, and progression of vascular lesions (20, 46). Our CFD analysis demonstrated markedly elevated OSI within the dissection, signifying severe flow disturbance. For the fenestration, the smaller channel exhibited higher OSI, but overall values remained lower than those in the dissection. The clinical significance of these OSI differences is twofold: in the dissection, elevated OSI supported the decision for aggressive intervention to prevent thromboembolic complications; in the fenestration, relatively lower OSI values provided reassurance that conservative management was appropriate.

The clinical application of CFD has increasingly been recognized as a valuable adjunct in cerebrovascular decision-making. For complex intracranial aneurysms, CFD-based frameworks have been developed to evaluate alternative surgical interventions by providing quantitative hemodynamic parameters for each option, enabling neurosurgeons to select the optimal strategy based on individualized flow, pressure, and WSS profiles (47, 48). In the context of ICA dissection, CFD has been successfully employed to assess postoperative hemodynamics and to identify patients at risk of downstream complications. More broadly, CFD has been integrated into the clinical management of various cerebrovascular conditions, including intracranial atherosclerotic stenosis, where translesional pressure gradients derived from CFD help distinguish between embolic and hypoperfusion stroke mechanisms, thereby guiding tailored blood pressure management (9). In our case, the combined assessment of elevated FP, bimodal WSS gradients, and high OSI in the dissection—contrasted with the uniformly low FP, stable WSS, and minimal OSI in the fenestration—provided objective, quantitative evidence that justified endovascular treatment for the dissection while deeming the fenestration hemodynamically stable. This decision was further supported by the patient's clinical symptoms (episodic dizziness) and the absence of thrombus or flow compromise in the fenestration on imaging.

Role of CFD in Elucidating Pathogenesis. Beyond clinical decision-making, CFD analysis can offer insights into the pathophysiological mechanisms underlying vascular anomalies. Disturbed flow patterns characterized by low WSS and high OSI have been shown to activate the endothelial NF-*κ*B pathway, sustaining chronic inflammation that may promote aneurysm initiation, growth, and rupture (16, 17). Similarly, CFD has been used to reveal mechanisms of thrombus formation in other vascular anomalies, such as carotid web, where preoperative CFD demonstrated blood stagnation and low WSS around the web structure, providing rheological evidence for the high thromboembolic risk associated with these lesions (10). In our case, the elevated OSI in both the dissection and the smaller fenestration channel suggests that even hemodynamically stable fenestrations may harbor regions of disturbed flow that could theoretically predispose to thrombus formation. However, in the absence of clinical symptoms or imaging evidence of thrombosis, conservative management with antiplatelet therapy appears sufficient. This observation underscores the importance of integrating CFD-derived hemodynamic data with clinical and imaging findings to guide individualized treatment decisions.

## 5 Conclusion

This case represents a rare bilateral heterogeneous vascular lesion, with a congenital developmental anomaly on one side and an acquired vascular lesion on the contralateral side. Such coexistence is clinically uncommon, with only a few reported cases. It suggests that the patient may have an underlying predisposition to congenital vascular wall weakness, and the risk of stroke may be higher than that associated with a single lesion, warranting increased clinical attention. Computational fluid dynamics (CFD) simulation intuitively demonstrates the hemodynamic characteristics of these two coexisting lesions, providing a more objective basis for disease risk assessment and treatment decision-making. This case serves as a valuable clinical research model for investigating the association between congenital cerebrovascular developmental defects and acquired vascular injury, as well as the interactive mechanisms of bilateral cerebral blood flow. Long-term follow-up will help improve our understanding of the pathogenesis and natural history of this condition, with significant implications for treatment decisions and stroke management.

## Data Availability

The raw data supporting the conclusions of this article will be made available by the authors, without undue reservation.

## References

[1] Miyake S Falzon A Kee TP Andrade H Krings T. Treatment of an intracranial aneurysm in the setting of fenestration of cranial division of the internal carotid artery: technical considerations and a literature review. Interv Neuroradiol. (2024) 17:15910199241262845. 10.1177/15910199241262845 PMC11571166 38881349

[2] Hasegawa T Kashihara K Ito H Yamamoto S. Fenestration of the internal carotid artery. Surg Neurol. (1985) 23(4):391–5. 10.1016/0090-3019(85)90214-9 3975829

[3] Nakamura H Yamada H Nagao T Fujita K Tamaki N. Fenestration of the internal carotid artery associated with an ischemic attack–case report. Neurol Med Chir (Tokyo). (1993) 33(5):306–8. 10.2176/nmc.33.306 7687037

[4] Gailloud P Carpenter J Heck DV Murphy KJ. Pseudofenestration of the cervical internal carotid artery: a pathologic process that simulates an anatomic variant. AJNR Am J Neuroradiol. (2004) 25(3):421–4. 10.1055/s-2003-814851 15037466 PMC8158555

[5] Park SH Lee CY. Supraclinoid internal carotid artery fenestration harboring an unruptured aneurysm and another remote ruptured aneurysm: case report and review of the literature. J Cerebrovasc Endovasc Neurosurg. (2012) 14(4):295–9. 10.7461/jcen.2012.14.4.295 23346545 PMC3543915

[6] Zheng M Liu X Song Y Zhang J Han J. Rare fenestration of an occluded internal carotid artery treated with stenting. J Vasc Surg. (2020) 72(1):319–20. 10.1016/j.jvs.2020.03.034 32553403

[7] Makino S Shimano K Shiratori S Nagano H Ujiie H. Influence of different outflow boundary conditions on hemodynamic analysis of cerebral aneurysm. J Biorheol. (2023) 37(1):21–34. 10.17106/jbr.37.21

[8] Tyfa Z Reorowicz P Obidowski D Jóźwik K. Influence of fluid rheology on blood flow haemodynamics in patient-specific arterial networks of varied complexity—in-silico studies. Acta Mech Autom. (2024) 18(1):1–12. 10.2478/ama-2024-0002

[9] Cebral JR Castro MA Appanaboyina S Putman CM Millan D Frangi AF. Efficient pipeline for image-based patient-specific analysis of cerebral aneurysm hemodynamics: technique and sensitivity. IEEE Trans Med Imaging. (2005) 24(4):457–67. 10.1109/TMI.2005.844159 15822804

[10] Hodis S Uthamaraj S Smith AL Dennis KD Kallmes DF Dragomir-Daescu D. Grid convergence errors in hemodynamic solution of patient-specific cerebral aneurysms. J Biomech. (2012) 45(16):2907–13. 10.1016/j.jbiomech.2012.07.030 23062796

[11] German Medical Science. Mesh resolution dependence of wall shear stress and oscillatory shear index in cerebral aneurysm CFD. 2020 – 71st annual meeting of the German society of neurosurgery. DGNC. 10.3205/20dgnc362

[12] Ford MD Alperin N Lee SH Holdsworth DW Steinman DA. Characterization of volumetric flow rate waveforms in the normal internal carotid and vertebral arteries. Physiol Meas. (2005) 26(4):477–88. 10.1088/0967-3334/26/4/013 15886442

[13] Van Doormaal JP Raithby GD. Enhancements of the SIMPLE method for predicting incompressible fluid flows. Numer Heat Transfer. (1984) 7(2):147–63. 10.1080/01495728408961817

[14] Kim HJ Vignon-Clementel IE Coogan JS Figueroa CA Jansen KE Taylor CA. Patient-specific modeling of blood flow and pressure in congenital heart disease. Int J Numer Method Biomed Eng. (2010) 26(10):1265–81. 10.1007/s10439-010-0083-6 20559732

[15] Ferziger JH Perić M. Computational Methods for Fluid Dynamics. 4th ed. Berlin/Heidelberg: Springer (2020). 10.1007/978-3-319-99693-6

[16] Takizawa K Bazilevs Y Tezduyar TE. Engineering analysis and medical applications of the variational multiscale method. In: Stein E de Borst R Hughes TJR, editors. Encyclopedia of Computational Mechanics. 2nd ed. Berlin/Heidelberg: Springer (2010).

[17] Takizawa K Bazilevs Y Tezduyar TE. Special techniques for wall shear stress and oscillatory shear index calculations in arterial flow simulations. Comput Mech (2011). 10.1007/978-90-481-3636-0_2

[18] Ku DN Giddens DP Zarins CK Glagov S. Pulsatile flow and atherosclerosis in the human carotid bifurcation. Positive correlation between plaque location and low oscillating shear stress. Arteriosclerosis. (1985) 5(3):293–302. 10.1161/01.atv.5.3.293 3994585

[19] Xiang J Natarajan SK Tremmel M Ma D Mocco J Hopkins LN. Hemodynamic-morphologic discriminants for intracranial aneurysm rupture. Stroke. (2011) 42(1):144–52. 10.1161/STROKEAHA.110.592923 21106956 PMC3021316

[20] Tang X Zhou L Wen L Wu Q Leng X Xiang J. Morphological and hemodynamic characteristics associated with the rupture of multiple intracranial aneurysms. Front Neurol. (2022) 12:811281. 10.3389/fneur.2021.811281 35126301 PMC8812485

[21] Bouthillier A van Loveren HR Keller JT. Segments of the internal carotid artery: a new classification. Neurosurgery. (1996) 38(3):425–32:discussion 432–3. 10.1097/00006123-199603000-00001 8837792

[22] Killien FC Wyler AR Cromwell LD. Duplication of the internal carotid artery. Neuroradiology. (1980) 19(2):101–2. 10.1007/BF00342603 7366832

[23] Tanaka M Matsumoto S. Title missing?—original list only had journal info. Neurol Med Chir (Tokyo). (1982) 22(4):291–4. 10.2176/nmc.22.291 6179001

[24] Chess MA Barsotti JB Chang JK Ketonen LM Westesson PL. Duplication of the extracranial internal carotid artery. AJNR Am J Neuroradiol. (1995) 16(7):1545–7. 7484653 PMC8338074

[25] Koenigsberg RA Zito JL Patel M Swartz JD Goldofsky E Zahtz G. Fenestration of the internal carotid artery: a rare mass of the hypotympanum associated with persistence of the stapedial artery. AJNR Am J Neuroradiol. (1995) 16(4 Suppl):908–10. 7611071 PMC8332278

[26] Mangla S Teitelbaum GP. Multichannel fenestrations of the petrous segment of the internal carotid artery. AJNR Am J Neuroradiol. (1999) 20(4):590–2. 10319967 PMC7056027

[27] Ahn JY Kim OJ Joo YJ Joo JY. Fenestration of the internal carotid artery associated with arterial dissections. J Clin Neurosci. (2003) 10(2):257–60. 10.1016/s0967-5868(02)00331-4 12637067

[28] Goto Y Yoshida K Oshimoto T Wataya T Hojo M Chin M. Fenestration of the extracranial internal carotid artery: report of two cases. No to Shinkei. (2003) 55(7):623–8. 12910998

[29] Chng SM Alvarez H Marsot-Dupuch K Mercier P Lasjaunias P. “Duplicated” or “multiple” cervical internal carotid and vertebral arteries from fenestration, duplication and vasa vasorum to segmental rete. Interv Neuroradiol. (2004) 10(4):301–7. 10.1177/159101990401000403 20587213 PMC3463289

[30] Kung DK Liu W Smoker WR Hasan DM. Duplication of the internal carotid artery presenting with severe atherosclerotic stenosis. J Clin Neurosci. (2011) 18(7):982–3. 10.1016/j.jocn.2010.11.026 21570303

[31] Hacein-Bey L Raghavan N Mukundan G Sekhon AK Dodrill LK Park TC. Fenestration of the petrous internal carotid artery with short segment duplication mimicking an arterial dissection. Clin Radiol. (2013) 68(9):972–5. 10.1016/j.crad.2013.04.015 23790690

[32] Liang Z Xiu C Liu Z Sun X Yu G Tao M. Misdiagnosis of a patient with internal carotid artery fenestration: a case report and literature review. Ann Vasc Surg. (2016) 30:309.e1–4. 10.1016/j.avsg.2015.07.031 26522583

[33] Mărginean L Filep RC Constantin C Bălaşa AF Mühlfay G. Fenestration of the cervical internal carotid artery misdiagnosed as dissection. Rom J Morphol Embryol. (2020) 61(1):257–60. 10.47162/RJME.61.1.30 32747919 PMC7728124

[34] Nasel C Poetsch A Brunner C Moser E. Transitory ischemic attack associated with a rare fenestration of the cervical segment of the internal carotid artery: a case report. J Med Case Rep. (2022) 16(1):13. 10.1186/s13256-021-03227-0 35031059 PMC8760826

[35] Wang JW Zhang Y Li X Zhan QL Yang YT Jin G. Internal carotid artery fenestration malformation resembling an arterial dissection in shape: report of one case. J Interv Radiol. (2024) 33(8):895–97.

[36] Koh EY Ocasio L Macedo T Keyhani A. Bilateral internal carotid artery fenestration. J Vasc Surg Cases Innov Tech. (2024) 10(5):101562. 10.1016/j.jvscit.2024.101562 39224694 PMC11367408

[37] Pentara NV Koutroulou I Finitsis S Rafailidis V Psoma E Grigoriadis N. Cervical internal carotid artery fenestration: a rare cause of lumen “dissection”. Surg Radiol Anat. (2024) 46(10):1659–62. 10.1007/s00276-024-03457-z 39136749

[38] Padget D. The development of the cranial arteries in the human embryo. Contrib Embryol. (1948) 32:205–61.

[39] Congdon ED. Transformation of the aortic-arch system during the development of the human embryo. Contrib Embryol. (1922) 14:47–110.

[40] Lie TA Hage J. Congenital anomalies of the carotid arteries. Plast Reconstr Surg. (1968) 42(3):163–9. 10.1097/00006534-196809000-000464874830

[41] Yamada T Inagawa T Takeda T. Ruptured aneurysm at the anterior cerebral artery fenestration. Case report. J Neurosurg. (1982) 57(6):826–8. 10.3171/jns.1982.57.6.0826 7143067

[42] Provenzale JM Sarikaya B Hacein-Bey L Wintermark M. Causes of misinterpretation of cross-sectional imaging studies for dissection of the craniocervical arteries. AJR Am J Roentgenol. (2011) 196(1):45–52. 10.2214/AJR.10.5384 21178045

[43] Suzuki T Stapleton CJ Koch MJ Tanaka K Fujimura S Suzuki T. Decreased wall shear stress at high-pressure areas predicts the rupture point in ruptured intracranial aneurysms. J Neurosurg. (2019) 132(4):1116–22. 10.3171/2018.12.JNS182897 30875692

[44] Boussel L Rayz V McCulloch C Martin A Acevedo-Bolton G Lawton M. Aneurysm growth occurs at region of low wall shear stress: patient-specific correlation of hemodynamics and growth in a longitudinal study. Stroke. (2008) 39(11):2997–3002. 10.1161/STROKEAHA.108.521617 18688012 PMC2661849

[45] Kulcsár Z Ugron A Marosfoi M Berentei Z Paál G Szikora I. Hemodynamics of cerebral aneurysm initiation: the role of wall shear stress and spatial wall shear stress gradient. AJNR Am J Neuroradiol. (2011) 32(3):587–94. 10.3174/ajnr.A2339 21310860 PMC8013095

[46] Meng H Tutino VM Xiang J Siddiqui A. High WSS or low WSS? Complex interactions of hemodynamics with intracranial aneurysm initiation, growth, and rupture: toward a unifying hypothesis. AJNR Am J Neuroradiol. (2014) 35(7):1254–62. 10.3174/ajnr.A3558 23598838 PMC7966576

[47] Perera R Isoda H Ishiguro K Mizuno T Takehara Y Terada M. Assessing the risk of intracranial aneurysm rupture using morphological and hemodynamic biomarkers evaluated from magnetic resonance fluid dynamics and computational fluid dynamics. Magn Reson Med Sci. (2020) 19(4):333–44. 10.2463/mrms.mp.2019-0107 31956175 PMC7809142

[48] Reorowicz P Obidowski D Klosinski P Szubert W Stefanczyk L Jozwik K. Numerical simulations of the blood flow in the patient-specific arterial cerebral circle region. J Biomech. (2014) 47(7):1642–51. 10.1016/j.jbiomech.2014.02.039 24674598

